# Key genes associated with non-alcoholic fatty liver disease and hepatocellular carcinoma with metabolic risk factors

**DOI:** 10.3389/fgene.2023.1066410

**Published:** 2023-03-06

**Authors:** Fan Yang, Beibei Ni, Qinghai Lian, Xiusheng Qiu, Yizhan He, Qi Zhang, Xiaoguang Zou, Fangping He, Wenjie Chen

**Affiliations:** ^1^ Department of Infectious Diseases, The First People’s Hospital of Kashi, The Kashi Affiliated Hospital, Sun Yat-Sen University, Kashi, China; ^2^ Biotherapy Centre, The Third Affiliated Hospital, Sun Yat-Sen University, Guangzhou, China; ^3^ Postdoctoral Research Station, Xinjiang Medical University, Ürümqi, China; ^4^ Cell-Gene Therapy Translational Medicine Research Centre, The Third Affiliated Hospital, Sun Yat-Sen University, Guangzhou, China; ^5^ Department of Hepatobiliary and Pancreatic Surgery, The Eighth Affiliated Hospital, Sun Yat-Sen University, Shenzhen, Guangdong, China

**Keywords:** hepatocellular carcinoma (HCC), metabolic risk factors (MRFs), non-alcoholic fatty liver disease (NAFLD), differentially expressed genes (DEGs), diagnosis, prognosis

## Abstract

**Background:** Hepatocellular carcinoma (HCC) has become the world’s primary cause of cancer death. Obesity, hyperglycemia, and dyslipidemia are all illnesses that are part of the metabolic syndrome. In recent years, this risk factor has become increasingly recognized as a contributing factor to HCC. Around the world, non-alcoholic fatty liver disease (NAFLD) is on the rise, especially in western countries. In the past, the exact pathogenesis of NAFLD that progressed to metabolic risk factors (MFRs)-associated HCC has not been fully understood.

**Methods:** Two groups of the GEO dataset (including normal/NAFLD and HCC with MFRs) were used to analyze differential expression. Differentially expressed genes of HCC were verified by overlapping in TCGA. In addition, functional enrichment analysis, modular analysis, Receiver Operating Characteristic (ROC) analysis, LASSO analysis, and Genes with key survival characteristics were analyzed.

**Results:** We identified six hub genes (FABP5, SCD, CCL20, AGPAT9(GPAT3), PLIN1, and IL1RN) that may be closely related to NAFLD and HCC with MFRs. We constructed survival and prognosis gene markers based on FABP5, CCL20, AGPAT9(GPAT3), PLIN1, and IL1RN.This gene signature has shown good diagnostic accuracy in both NAFLD and HCC and in predicting HCC overall survival rates.

**Conclusion:** As a result of the findings of this study, there is some guiding significance for the diagnosis and treatment of liver disease associated with NAFLD progression.

## 1 Introduction

Hepatocellular carcinoma (HCC) accounts for about 90% of all liver cancers, making it the second leading cause of cancer death worldwide (Llovet et al., 2021, M. A; Morse et al., 2019). The development of HCC usually follows a background of chronic low-grade inflammation characterised by chronic liver damage followed by inflammation, hepatocellular necrosis, and regeneration. HCCs are predominantly caused by Hepatitis B (HBV), Hepatitis C (HCV) infection, alcohol consumption, as well as metabolic perturbations leading to non-alcoholic fatty liver disease (NAFLD) (R. E. Ericksen et al., 2019, C; Trierweiler et al., 2016). Due to universal vaccination and antiviral therapy, viral HCC prevalence is decreasing. It will be necessary to modify strategies for cancer prevention, prediction, and surveillance for HCC (Awosika and Sohal, 2022, S. F; Huang et al., 2018). The metabolic syndrome refers to a group of disorders that include dyslipidemia, hyperglycemia, and obesity, has received increasing attention as a novel risk factor for HCC (K. Akahoshi et al., 2016). There was a greater than 100-fold increase in risk for HBV or HCV carriers who also had diabetes or obesity, which suggests synergistic effects of metabolic factors and hepatitis (C. L. Chen et al., 2008). Patients with metabolic risk factors (MRFs) may be at greater risk of developing hepatocarcinogenesis when FABP4 is overexpressed in HSCs (N. Chiyonobu et al., 2018).

According to statistics, 25 per cent of the population worldwide suffers from NAFLD (A. Lonardo et al., 2016). NAFLD is becoming more prevalent worldwide, especially in western countries (Mancina et al., 2016). Consequently, NAFLD has become an economic and health concern worldwide. There is no doubt that NAFLD is a hepatic manifestation of metabolic syndrome (MS) and is often associated with dyslipidemia, obesity, and T2DM (J. Wattacheril, 2020). As a result of liver lipid accumulation, NAFLD can cause inflammation and damage to the hepatocytes (J. Wattacheril, 2020). The liver biopsy usually shows milder forms (steatosis) to severe conditions (non-alcoholic steatohepatitis (NASH), advanced fibrosis, cirrhosis) (Pouwels et al., 2022).

In the past, the exact pathogenesis of NAFLD that progressed to MFRs-associated HCC has not been fully understood. High-throughput gene chips and transcriptome sequencing have entirely changed the previous systematic analysis methods for disease research (M. Bustoros et al., 2020). RNA sequencing and high-throughput microarrays help to identify reliable biological markers, classify diseases, and reveal mechanisms of disease development. The discovery of new biomarkers can be helpful in predicting risk and determining which treatment is most suitable for an individual patient. Thus, the prediction of candidate genes may also be based on NAFLD-HCC with MRFs pathogenesis.

This study aims to identify the key genes involved in NAFLD and HCC with MRFs and to provide a reference for further study of the transformation of MFRs-associated HCC and a molecular-targeted approach to cancer treatment. In this study, we analyzed microarray data comprehensively, selecting normal tissues and NAFLD samples and microarray data of MFRs-associated HCC and adjacent normal tissues, and separately analysed the differentially expressed genes (DEGs) in both groups of chips. Combining the GEO DEG data of human HCC with MFRs and normal liver tissue with chip data to determine key DEGs that directly affect the diagnosis and treatment of NAFLD. Afterwards, further functional enrichment analysis was conducted to determine how DEGs regulate the main biological functions. Furthermore, by using protein-protein interaction (PPI) networks and survival analysis of patient data, key genes are identified that affect the diagnosis, treatment, and prognosis of patients with NAFLD.

## 2 Methods

### 2.1 Profiles of gene expression

GSE63067, GSE89632, and GSE102079 datasets were downloaded from Gene Expression Omnibus (GEO), an open-access database that provides gene expression profiles. GSE63067 (Frades et al., 2015) and GSE102079 (N. Chiyonobu, S. Shimada, Y. Akiyama, K. Mogushi, M. Itoh, K. Akahoshi, S. Matsumura, K. Ogawa, H. Ono, Y. Mitsunori, D. Ban, A. Kudo, S. Arii, T. Suganami, S. Yamaoka, Y. Ogawa, M. Tanabe and S. Tanaka, 2018) are both based on the GPL570 [(HG-U133_Plus_2) Affymetrix Human Genome U133 Plus 2.0 Array]. GSE89632 (B. M. Arendt et al., 2015) is based on [(GPL14951) Illumina HumanHT-12 WG-DASL V4.0 R2 expression bead chip]. The title of the GSE63067 data set is “Expression data from human non-alcoholic fatty liver disease stages”. The data contained the gene expression profiles of 11 NAFLD patients and seven non-NAFLD controls. The title of the GSE102079 data set is “FABP4 overexpressed in intratumoral hepatic stellate cells within hepatocellular carcinoma with metabolic risk factors”. Between 2006 and 2011, 152 patients who underwent curative hepatic resection for HCC at Tokyo Medical and Dental University Hospital participated in an integrated gene expression microarray study. In the control group, 14 adjacent liver tissues were obtained from patients with metastases of colorectal cancer without chemotherapy. The validation data set was from GSE89632 and The Cancer Genome Atlas (TCGA) data set. The title of the GSE89632 data set is “Genome-wide analysis of hepatic gene expression in patients with non-alcoholic fatty liver disease and healthy donors with hepatic fatty acid composition and other nutritional factors”. A cross-sectional study included 20 patients with simple steatosis (SS), 19 non-alcoholic steatohepatitis (NASH), and 24 healthy liver donors. The TCGA database of liver hepatocellular carcinoma (LIHC) contains RNA-Seq data for 374 HCC patients and 50 normal tissues (https://portal.gdc.cancer.gov/) for gene expression and immune system infiltrates.

### 2.2 Analysis of differentially expressed genes (DEGs) in NAFLD and HCC with MRFs

A comparison of DEGs between NAFLD and normal controls, HCC patients with MRFs, and corresponding controls was performed using the limma R package“complexheatmap” and “ggplot2” to generate heat maps and volcano maps, respectively, which is an efficient analysis method in bioinformatics (M. E. Ritchie et al., 2015). In NAFLD datasets, the selected criteria were *p*-value <0.05 and |log2FC|>1. In HCC datasets, the selected criteria were *p*-value <0.05 and |log2FC|>1. Additionally, the overlapping DEGs between NAFLD and HCC with MRFs were determined by Venn diagrams using the Venn platform (http://bioinformatics.psb.ugent.be/webtools/Venn/). A subsequent analysis was performed on these overlapping DEGs.

### 2.3 Functional classification and pathway enrichment for DEGs

GO function enrichment analyses were conducted on the above overlapping DEGs. It consisted of biological process (BP), cellular component (CC), and molecular function (MF) (2006). The analysis of KEGG signaling pathway enrichment using a package called “clusterProfiler” (M. Kanehisa et al., 2016). GO terms and KEGG pathways enriched with adjusted *p*-value of 0.05 were selected for analysis.

### 2.4 Establishment of protein-protein interactions and identification of hub genes

In order to further investigate the interactions between the above-mentioned common genes, a search tool called the Search Tool for Retrieval of Interacting Genes (STRING) has been developed for PPI network construction (D. Szklarczyk et al., 2015). Interaction scores of at least 0.4 were considered significant. Subsequently, PPI network visualisation was conducted with Cytoscape software. Then, the Maximal Clique Centrality (MCC), Density of Maximum Neighborhood Component (DMNC), Maximum Neighborhood Component (MNC), Degree, and Edge Percolated Component (EPC), algorithms in the cytoHubba plug-in (http://hub.iis.sinica.edu.tw/cytohubba/) was applied to identify PPI hub genes with high connectivity.

### 2.5 Comparing the hub gene expression degree and analysing the prognosis

Based on the TCGA database, the six hub genes expression in HCC normal tissues and tumor tissues was investigated. In LIHC, 374 HCC specimens with normal adjacent tissues and HCC tissue (50 each) were compared with neighbouring normal tissues. GEPIA was used to investigate the prognostic significance of hub genes (http://gepia.cancer-pku.cn/index.html) (Z. Tang et al., 2017). Survival analyses were considered significant when log-rank *p* < 0.05 was used.

### 2.6 Developing signatures and evaluating their reliability

Based on the training dataset, hub genes associated with prognosis were identified and assessed against other datasets for their predictive performance. Half of TCGA is set as the training set. The other half of TCGA is set as a validation set. The entire TCGA cohort is a verification set. Using univariate Cox proportional hazard regression analysis, it was evaluated whether hub genes are associated with overall survival (OS) in the training set. In the “glmnet” package, the Latent Selection Operator penalised Cox proportional hazard regression using Cox proportional hazards models. A prediction formula for gene characteristics was devised. The formula for the model is as follows: risk score = gene1×*β*1 (gene one expression level) + gene 2×*β*2 (gene two expression level) +…gene n×βn (gene n expression level). In this formula, genes are combined with gene expression values and regression coefficients from multiple Cox proportional hazards regression models (George et al., 2014; Zhang J. et al., 2021). Using the Kaplan–Meier (K–M) survival curves, survival comparisons were performed between low- and high-risk groups *via* the R package “survival”. Furthermore, a time-dependent receiver operating characteristic (ROC) analysis (including 1-, 3-, and 5-year survival) was conducted to evaluate hub gene sensitivity and specificity using the R package “survival ROC” (P. J. Heagerty et al., 2000). It is critical to consider the area of the AUC curve when trying to predict clinical outcomes. Prognosis is better when AUC >0.5; the closer AUC is to 1, the better.

### 2.7 The expression of hub genes is correlated with the presence of immune cells in tumor

Tumor contains a large number of immune cells, and the prognosis of high-grade HCC patients with high subtype of dominant immunity is obviously better (Y. Kurebayashi et al., 2018). To examine whether the expression of hub genes is correlated with the presence of immune cells in HCC, we examined the correlation between hub gene mRNA expression and tumor-infiltrating immune cells. The web tool TIMER was used (https://cistrome.shinyapps.io/timer/) (T. Li et al., 2017). Six tumor-infiltrating cell subsets were analysed, such as B cells, CD8^+^ T cells, CD4^+^ T cells, macrophages, neutrophils, and dendritic cells.

### 2.8 Statistical analysis

ROC curves for hub genes were constructed using the pROC package (X. Robin et al., 2011). To measure the effectiveness of the model, we calculated the area under the curves (AUC). These results showed the usefulness of genes for diagnostic purposes. *p*-values <0.05 were considered statistically significant.

## 3 Results

### 3.1 Identification of DEGs in NAFLD and HCC

**FIGURE 1:**
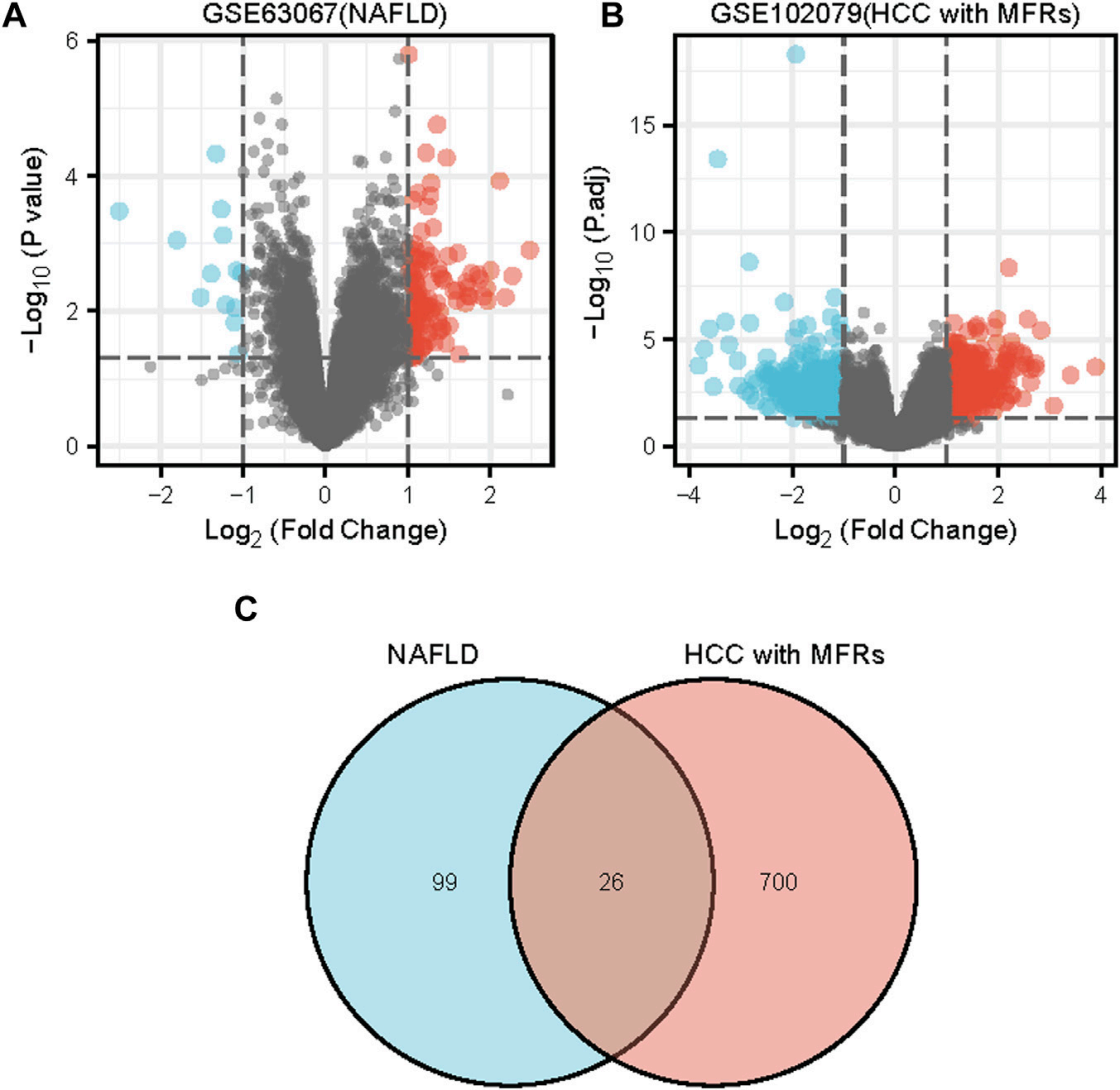
Differentially expressed genes (DEGs) shown in a volcano plot and Venn diagram **(A)** An analysis of the differential genes in GSE63067 using a volcano map. **(B)** An analysis of the differential genes in GSE102079 using a volcano map. **(C)** Venn diagram of DEGs in GSE63067 and GSE102079 data sets. Abbreviations: DEGs, differentially expressed genes; NAFLD, non-alcoholic fatty liver; HCC, Hepatocellular Carcinoma, MFRs, metabolic risk factors.

**FIGURE 2:**
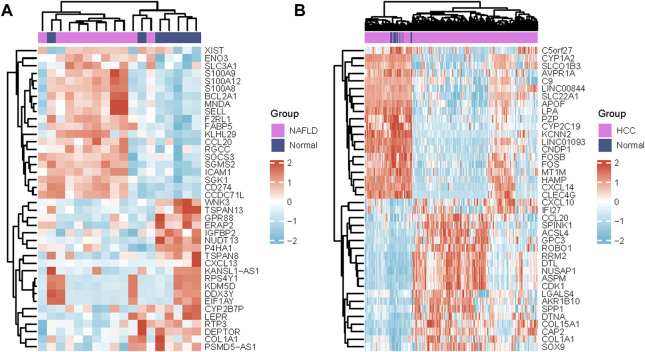
Differentially expressed genes heatmaps **(A)** Heat map of DEGs in GSE63067 (NAFLD) and **(B)** Heatmap of DEGs in GSE102079(HCC with MFRs). DEGs in red indicate upregulation, DEGs in blue indicate downregulation, and DEGs in white indicate no significant changes. Abbreviations: NAFLD, non-alcoholic fatty liver; HCC, Hepatocellular Carcinoma.

The series GSE63067 dataset about NAFLD and the series GSE102079 dataset about HCC from the NCBI GEO database was downloaded. Based on a *p*-value of 0.05 and |log2FC | of >1.0, 125 DEGs were identified in GSE63067, and 726 DEGs were identified in GSE102079 using the “limma” package in R software. Volcano plots and heatmaps were used to visualise the DEGs of the two data sets shown in Figures 1A,B and Figures 2A,B, respectively. Using the Venn Diagram online tool, 26 common genes of two diseases were identified and are shown in Figure 1C.

### 3.2 Analysis of pathways and functional roles associated with overlapping DEGs

**FIGURE 3:**
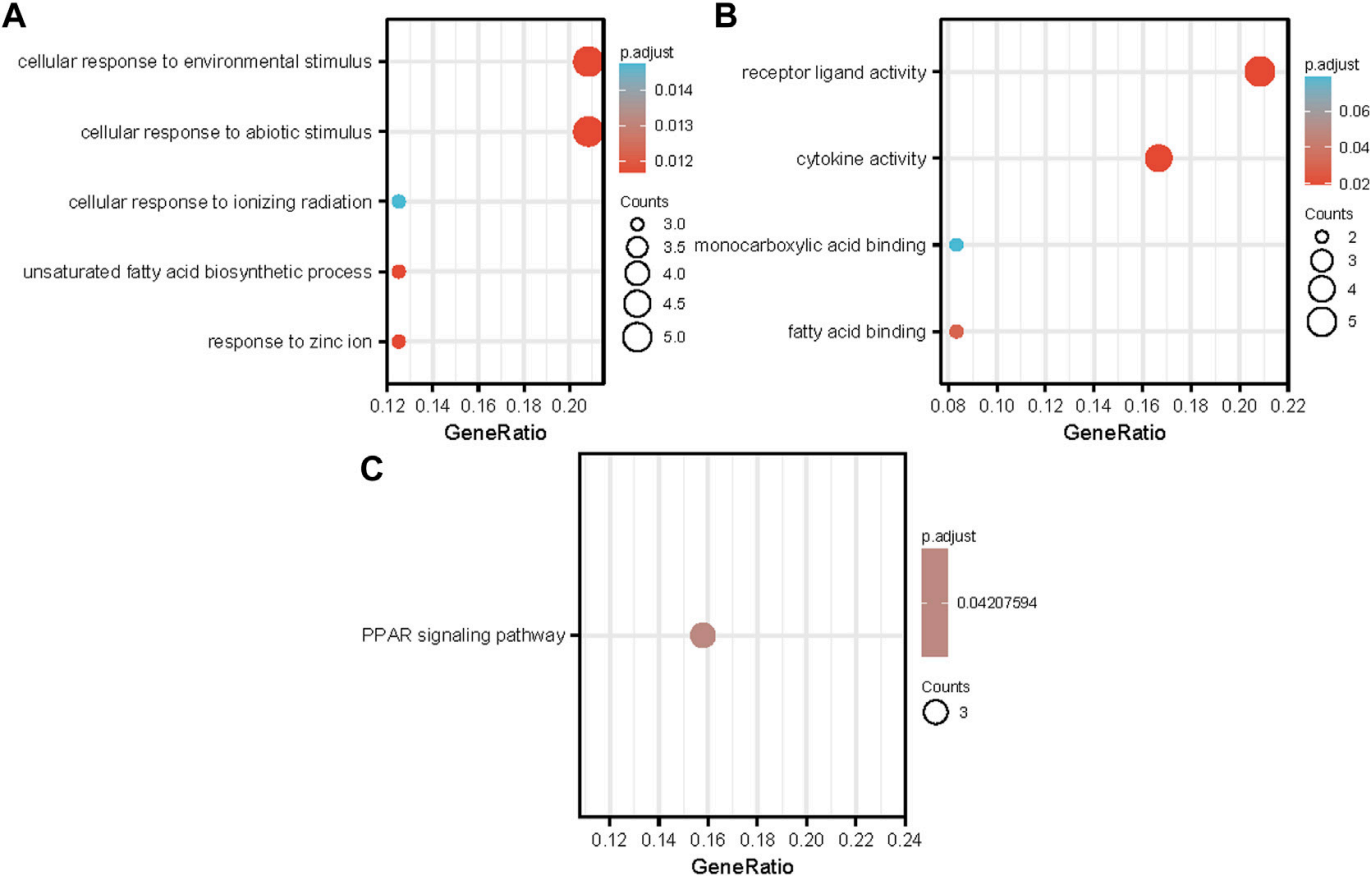
Analyses of functional enrichment between two groups of DEGs. **(A)** Enrichment results for GO biological processes; **(B)** Enrichment results for GO molecular function processes; **(C)** Enrichment results for KEGG pathways A bubble’s size represents the number of genes associated with each term. A term’s bubble size represents how many genes are associated with it. Each bubble’s color indicates the adjusted *p*-value abbreviations: GO, Gene Ontology; BP, biological process; MF, molecular function; KEGG, Kyoto Encyclopedia of Genes and Genomes.

Functional enrichment and KEGG pathway analyses of 26 common NAFLD and HCC genes were performed at a threshold of *p*-value <0.05. The results showed that DEGs were enriched in biological processes, including cellular response to environmental stimuli, cellular response to abiotic stimuli, cellular response to ionising radiation, unsaturated fatty acid biosynthetic process, and response to zinc ions (Figure 3A). Regarding molecular function, DEGs were principally associated with receptor ligand activity, cytokine activity, monocarboxylic acid binding, and fatty acid binding Figure 3B. The KEGG pathways of DEGs were enriched in the PPAR signalling pathway (Figure 3C).

### 3.3 Analysing the PPI network and selecting hub genes

**FIGURE 4:**
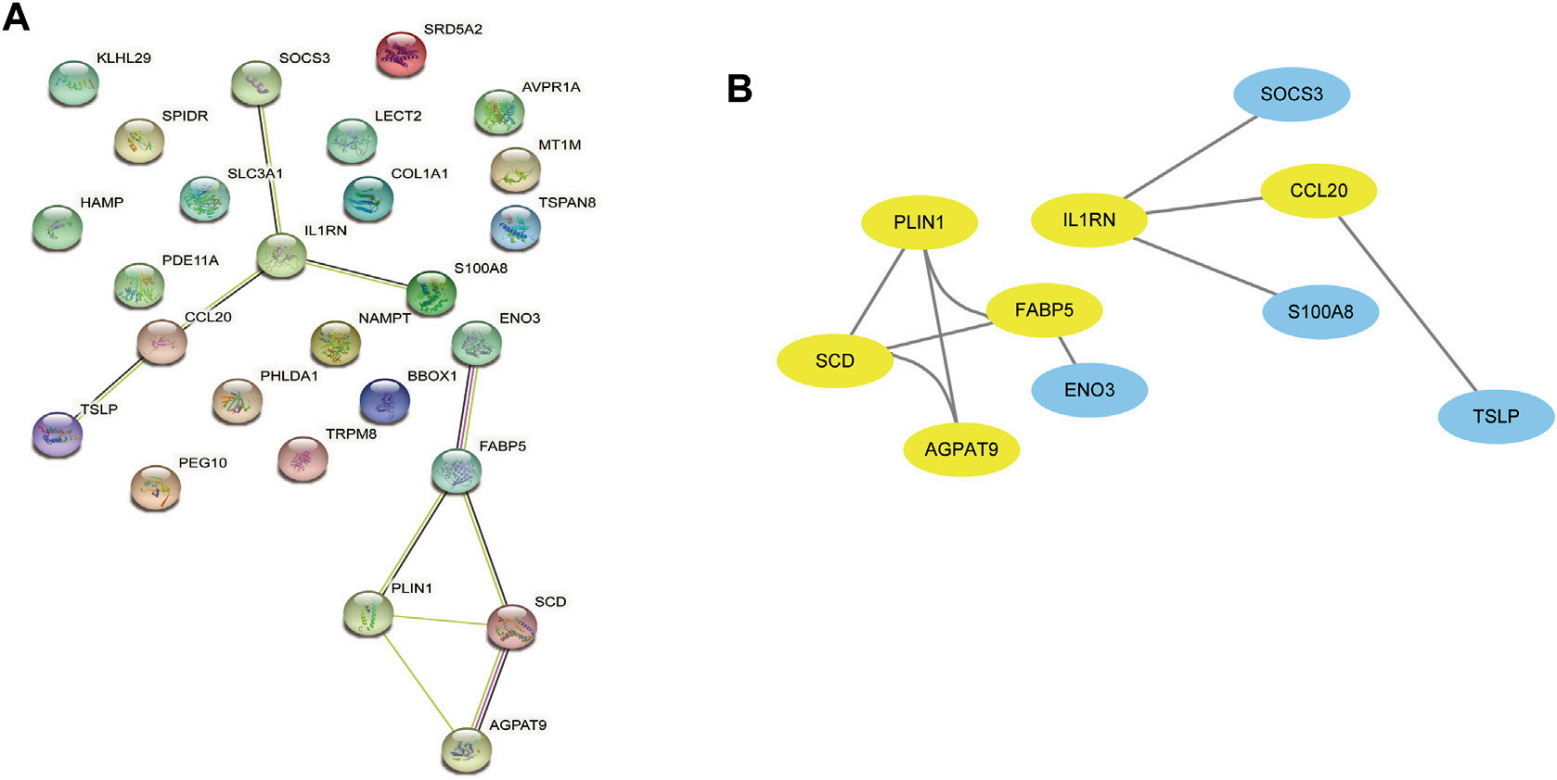
The PPI network analysis highlights the most significant modules related to DEGs. **(A)** 26 DEGs were used in the construction of this PPI network. **(B)** The most significant module of the PPI network includes 6 hub genes(yellow circles). DEGs differentially expressed genes; PPI, Protein-Protein interaction.

The PPI network was first performed based on the STRING database to investigate how DEGs interact with one another. Afterwards, the results were imported into Cytoscape to be analysed (Figure 4A). A Cytoscape plug-in, Cytohubba, was used to analyse the PPI network and identify hub genes. We got top 10 genes from protein-protein network ranked by five different algorithms of cytohubba including MCC, DMNC, MNC, Degree, and EPC (Table1). In this study, the genes with the top six values were considered as hub genes. Based on the five algorithms, the top six genes were determined to be hub genes: FABP5, SCD, CCL20, AGPAT9, PLIN1, IL1RN (Figure 4B).

**TABLE 1:** Results for analysis by Cytohubba.

node_name	MCC	DMNC	MNC	Degree	EPC
S100A8	1	0	1	1	1.947
SOCS3	1	0	1	1	1.998
FABP5	3	0.30779	2	3	2.985
ENO3	1	0	1	1	2.051
TSLP	1	0	1	1	1.853
IL1RN	3	0	1	3	2.56
CCL20	2	0	1	2	2.318
SCD	4	0.30898	3	3	3.027
PLIN1	4	0.30898	3	3	3.055
AGPAT9	2	0.30779	2	2	2.75

MCC, maximal clique centrality; DMNC, density of maximum neighborhood component; MNC, maximum neighborhood component; EPC, edge percolated component

### 3.4 The diagnostic value of hub genes has been validated

**FIGURE 5:**
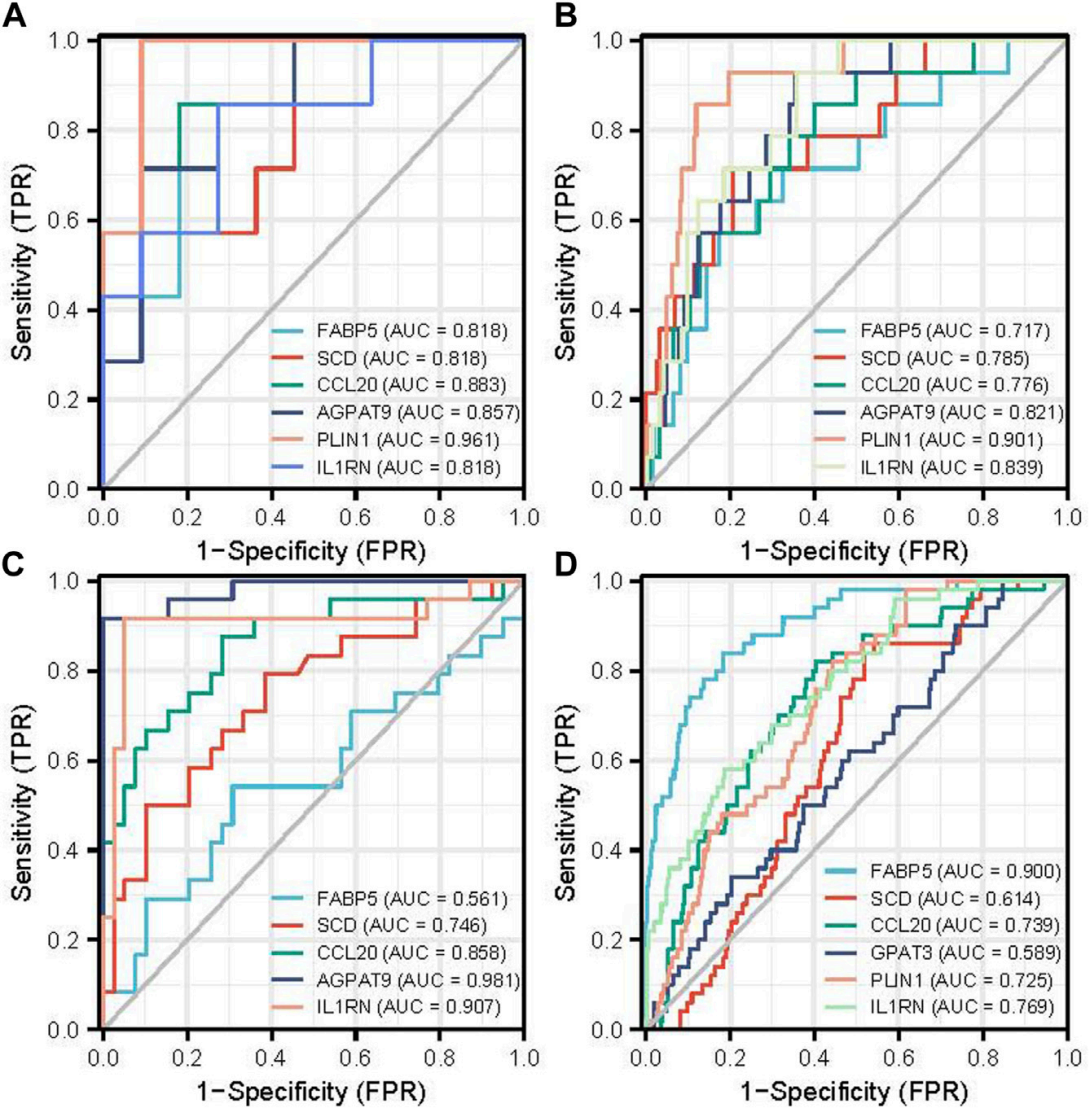
The diagnostic value of the top six hub genes with ROC curves in NAFLD and HCC. **(A)** The diagnostic value of the top six hub genes on ROC curves in NAFLD is based on the GSE63067 data set. **(B)** The diagnostic value of the top six hub genes on ROC curves in HCC with metabolic risk factors is based on the GSE102079 data set. **(C)** The diagnostic value of the top six hub genes on ROC curves in NAFLD is based on GSE89632 for validation. **(D)** The diagnostic value of the top six hub genes on ROC curves in HCC is based on TCGA data. Abbreviations: TPR, True Positive Rate: FPR, False Positive Rate.

To evaluate the diagnostic value of the top six hub genes obtained from the above analysis, ROC curves were constructed and their corresponding area under the curve (AUC) was calculated. Figure 5A shows the result of NAFLD. The AUC for FABP5, SCD, CCL20, AGPAT9, PLIN1, and IL1RN in NAFLD patients and normal controls were 0.828, 0.818, 0.883, 0.857, 0.961, 0.818 at NAFLD GSE63067 dataset. Figure 5B shows the ROC curves in HCC patients and normal controls. The AUC for FABP5, SCD, CCL20, AGPAT9, PLIN1, and IL1RN in HCC and the normal controls were 0.717, 0.785, 0.776, 0.821, 0.901, 0.839 at HCC with MRFs GSE102079 dataset. For validation, The AUC for FABP5, SCD, CCL20, AGPAT9, and IL1RN were 0.561, 0.746, 0.858, 0.981, 0.907 in NAFLD based on GSE89632 (Figure 5C). The AUC for FABP5, SCD, CCL20, AGPAT9, PLIN1, and IL1RN in HCC and the normal controls were 0.900, 0.614, 0.807, 0.589, 0.725, 0.769 at HCC TGCA dataset Figure 5D).

### 3.5 An evaluation of the expression patterns and survival analysis of six hub genes

**FIGURE 6:**
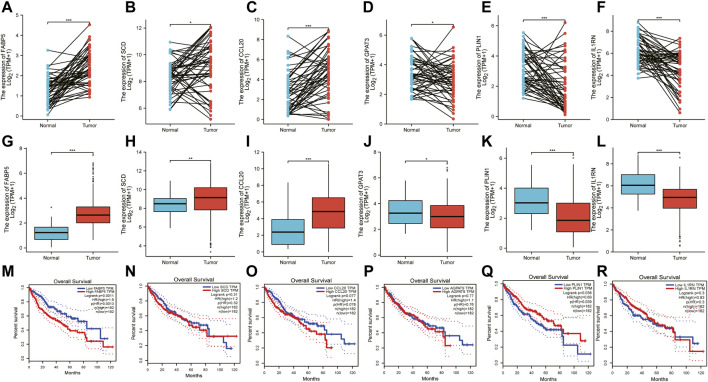
TCGA database, has been used to validate the expression patterns of six hub genes. **(A–F)** The different expressions of six hub genes in paired HCC and normal controls in TCGA-LIHC. **(G–L)** The different expressions of six hub genes between HCC and the normal group. **(M–R)** This Kaplan-Meier plot shows how the hub genes were significant prognostic factors.

Consistent with the results in GEO datasets, the mRNA expression of AGPAT9, PLIN1, and IL1RN was significantly downregulated. At the same time, that of FABP5, SCD, and CCL20 were upregulated considerably in TCGA HCC compared with non-tumor tissues (Figures 6A–L). According to the GEPIA web tool, FABP5 and PLIN1 mRNA expression are significantly linked to overall survival (OS) (Figures 6M–Q).

### 3.6 An analysis of the correlation between hub gene expression levels

**FIGURE 7:**
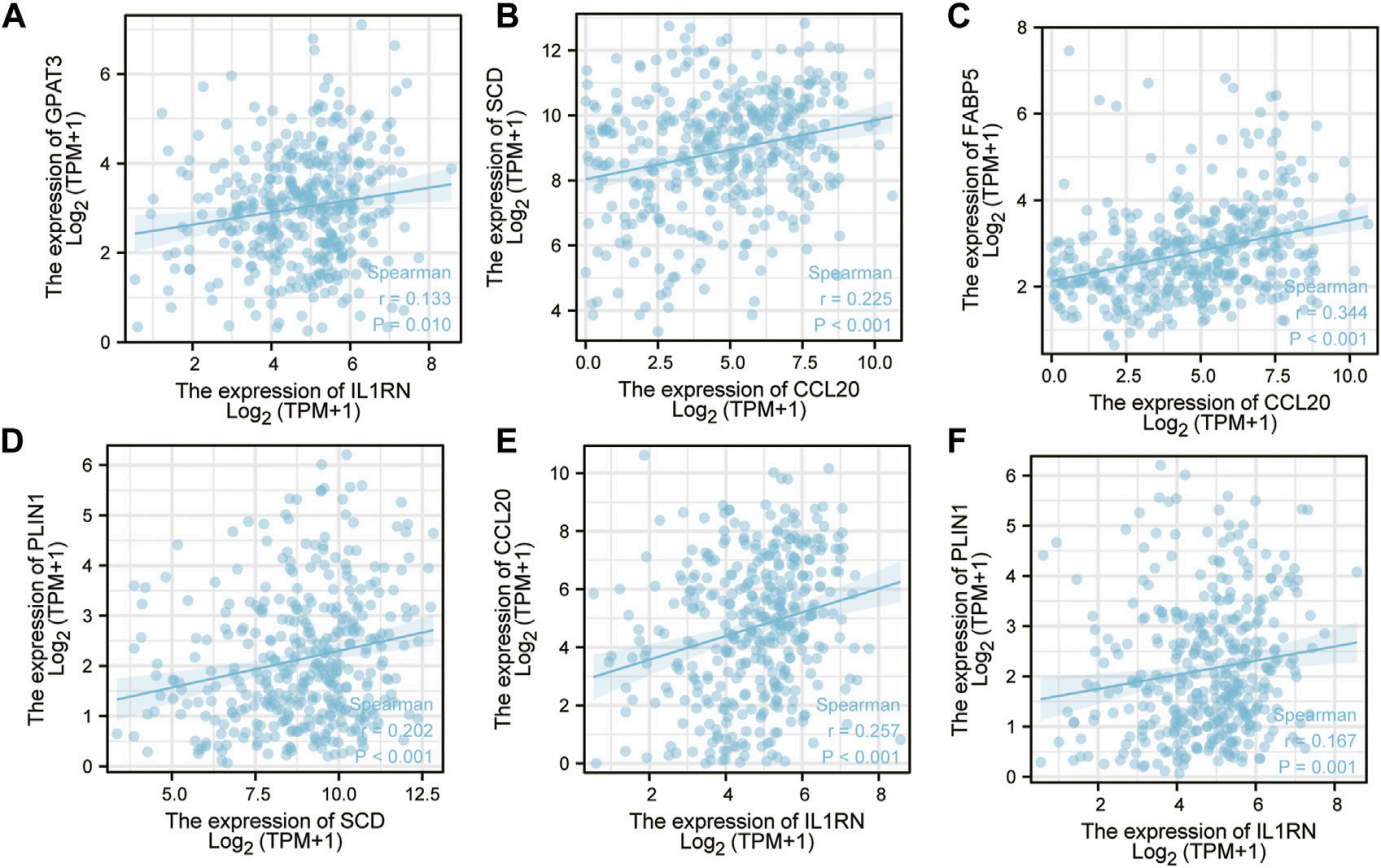
A correlation analysis was conducted on six key genes. **(A)** IL1RN-GPAT3 **(B)** CCL20-SCD **(C)** CCL20-FABP5 **(D)** SCD-PLIN1 **(E)** IL1RN-CCL20 **(F)** IL1RN-PLIN1.

The correlation of expression levels of hub genes was captured using GEPIA. An analysis of correlation was performed on any two of FABP5, SCD, CCL20, AGPAT9(GPAT3), PLIN1, and IL1RN and six hub genes. The above data indicate that upregulation of one of them will decrease the high expression of other genes. IL1RN and GPAT3 (Figure 7A), CCL20 and SCD (Figure 7B), CCL20 and FABP5 (Figure 7C), SCD and PLIN1 (Figure 7D), IL1RN and CCL20 (Figure 7E), and IL1RN and PLIN1 (Figure 7F) are all positively related to each other. This may indicate that there is a common transcription factor as well as epigenetic modifications controlling them all.

### 3.7 The construction of the hub gene prognostic signature

**FIGURE 8:**
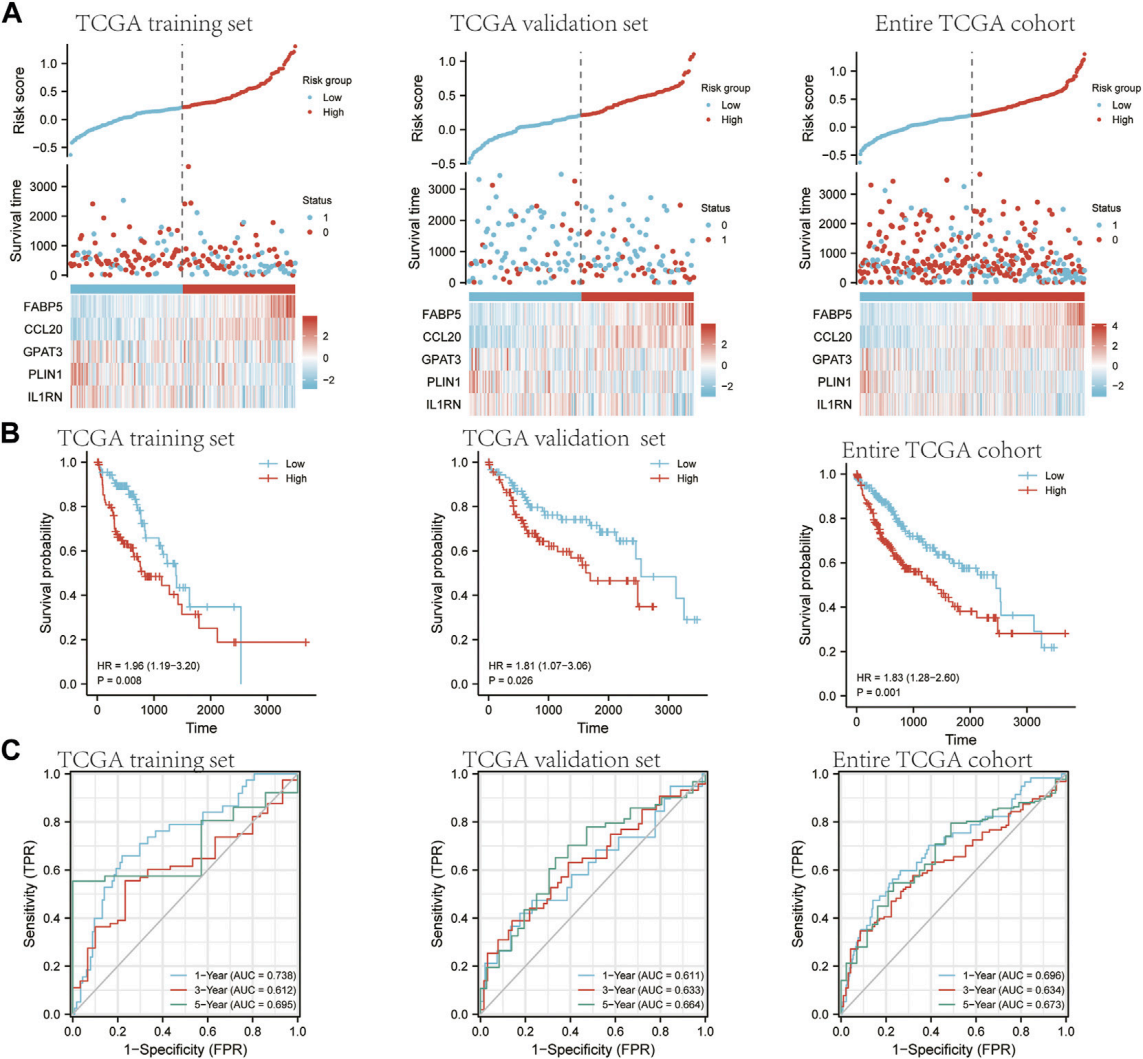
An analysis of the five-gene signature model in the TCGA cohort for prognosis. Half of TCGA is set as the training set. The other half of TCGA is designated as a validation set. The entire TCGA cohort is a verification set. **(A)** A comparison of risk score distribution, survival rates, and gene expression between patients in low- and high-risk groups in TCGA training set and TCGA validation set, entire TCGA cohort. **(B)** The Kaplan-Meier curves of OS for high-risk and low-risk groups in TCGA training set, TCGA validation set, and the entire TCGA cohort. **(C)** Time-dependent ROC curve AUCs from the TCGA training set, TCGA validation set, and entire TCGA cohort.

To avoid overfitting by LASSO regression, lambda. Min was selected, resulting in a more accurate prediction rate. We used the multivariate Cox proportional hazards regression analysis. Five prognostic genes were developed, including ABP5, CCL20, GPAT3, PLIN1, and IL1RNIn order to calculate the risk score for each patient, the following formula was used: risk score = (0.170124209 ×FABP5) + (0.073621309 ×CCL20) + (0.011005683 × GPAT3) + (−0.056212587 ×PLIN1)+ (−0.100588077 ×IL1RN). The LASSO coefficient for SCD is equal to 0. Hub gene risk scores were used to determine whether HCC patients were low-risk or high-risk (Figure 8A). A significantly worse OS was observed in high-risk patients compared to low-risk patients (Figure 8B, training set *p* = 0.008, validation set *p* = 0.0026, entire TCGA set *p* = 0.001). The reliability of hub genes was subsequently assessed using time-dependent ROC curves (Figure 8C). As a result, the area under the curve (AUC) was 0.738, 0.612, and 0.695 for 1-year, 3-year, and 5-year survival, respectively for the training set. The AUC was 0.611, 0.633, and 0.664 for 1-year, 3-year, and 5-year survival, respectively, for the validation set. These curves were also applied in the entire TCGA set. The AUC was 0.696, 0.634, and 0.673 for 1-year, 3-year, and 5-year survival, respectively.

### 3.8 Correlation analysis of hub gene mRNA levels with tuours-infiltrating immune cells

**FIGURE 9:**
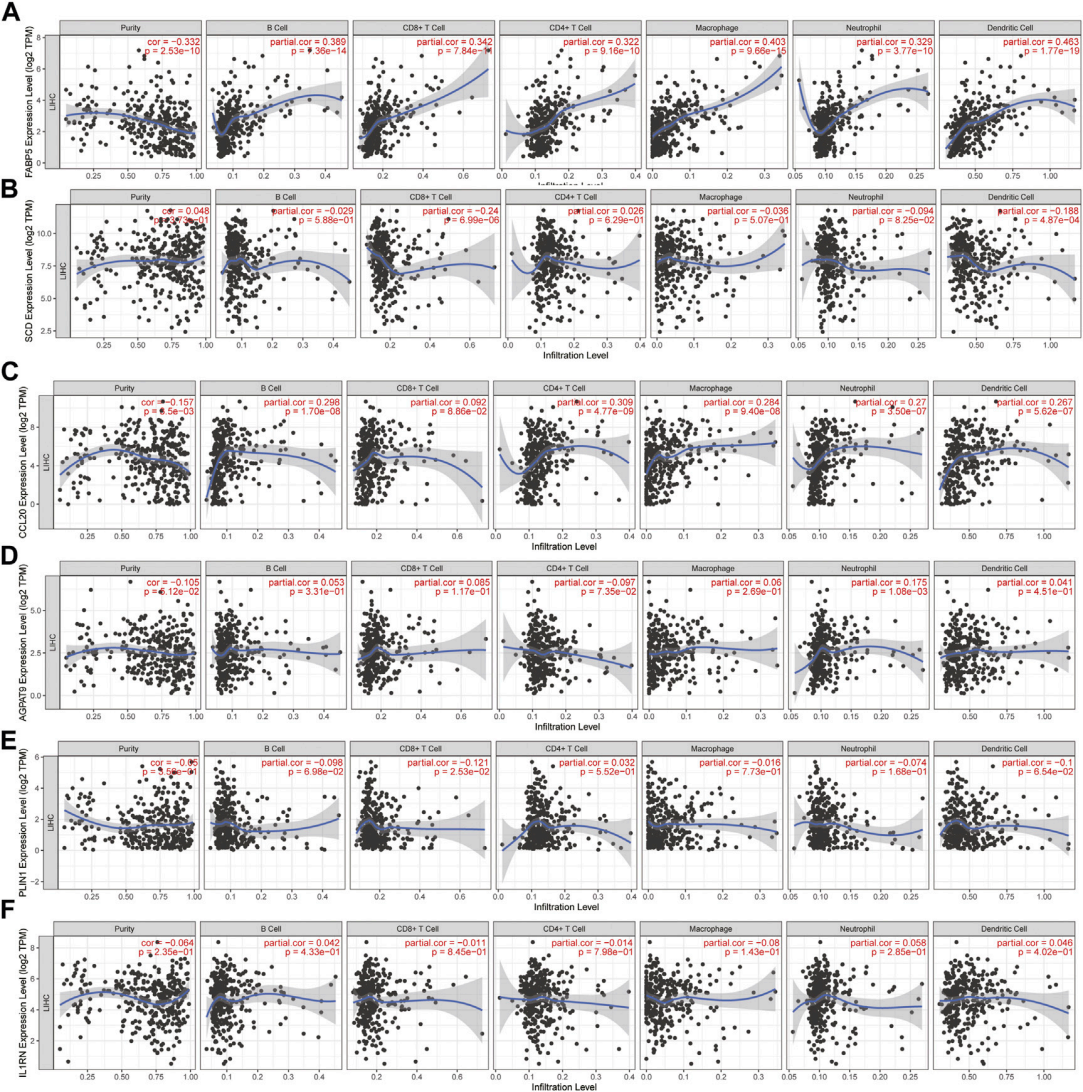
Correlation analysis of hub gene mRNA levels with tumor-infiltrating immune cells. **(A–F)** The correlation of FABP5, SCD, CCL20, AGPAT9, PLIN1, IL1RN mRNA with tumor-infiltrating immune cells. TIMER is the database used for the data (https://cistrome.shinyapps.io/timer/).

There are three kinds of cells in the tumor microenvironment: tumor cells, stromal cells, and immune cells that infiltrate the tumor. The TIMER web tool showed that the expression of all six hub genes was associated with infiltrating immune subsets, and the expression of NAFLD and HCC showed the most significant correlation with them. For B-cells, CD4^+^ T-cells, CD8^+^ T-cells, neutrophils, macrophages, and dendritic cells, the expression of FABP5 showed the most significant correlation with them (Figures 9A–F).

## 4 Discussion

In recent years, increasingly studies have confirmed the link between NAFLD and HCC. A higher risk of HCC has been associated with metabolic syndrome (Agosti et al., 2018, Y. P; Lin et al., 2022, Y; Tan et al., 2019). In the clinic, development and transformation of NAFLD are governed by common law, and its transformation process is also typical of HCC transformation. So far, the mechanism linking NAFLD and HCC remains unclear. Therefore, exploring the molecular mechanisms between NAFLD and other diseases and early identifying and intervening are likely to have significant clinical significance. Bioinformatics analyses comprehensively concentrate primarily on DEGs screening, the development of related protein interaction networks, the screening of genes, and the study of gene associations.

In this study, through searching the datasets of NAFLD and HCC with MRFs from the GEO database, we found 26 common DEGs between these diseases. The results of GO enrichment analysis indicated that the DEGs were mainly enriched in receptor-ligand activity, cytokine activity, monocarboxylic acid binding, and fatty acid binding. Based on the KEGG pathway enrichment analysis results, overlapping differential genes are mainly involved in the PPAR signalling pathway. As members of the nuclear receptor superfamily, PPARs can regulate multiple metabolic pathways and are effective targets in the treatment of many metabolic disorders, including NAFLD (Wu et al., 2021). The PPAR signalling pathway is critical to the progression of non-alcoholic steatohepatitis (Zhang Y. et al., 2021). It is possible to predict HCC prognosis using the PPAR signaling pathway effectively, independently, and usefully (Xu et al., 2021).

As a result of the PPI network and module analysis, we identified six key genes, including FABP5, SCD, CCL20, AGPAT9(GPAT3), PLIN1, and IL1RN. The six genes were all changed in both NAFLD patients and HCC patients with MRFs, suggesting that they may play an essential role in NAFLD and HCC with MRFs. An analysis of ROC curves was performed to validate the diagnostic value of NAFLD and HCC. This gene signature has shown good diagnostic accuracy in both NAFLD and HCC. The expression of FABP5 in NAFLD correlates with histological progression and the loss of hepatic fat during cirrhosis progression in NASH (K. Enooku et al., 2020). Several studies have shown that (fatty acid binding protein 5, FABP5) is highly expressed in HCC. It has been shown that FABP5 promotes angiogenesis and activates the IL6/STAT3/VEGFA pathway in HCCs (F. Liu et al., 2020). Overall survival time for HCC patients was negatively correlated with FABP5 levels in monocytes. The FABP5 protein promotes immune tolerance in patients with HCC by regulating monocytes and tumor-associated monocytes’ fatty acid oxidation process *via* suppressing the PPARα pathway (J. Liu et al., 2022). Our results indicated that FABP5 expression is significantly linked to the overall survival of HCC patients. FABP5 showed the most significant correlation with tumor-infiltrating immune subsets, such as B-cells, CD4^+^ T-cells, CD8^+^ T-cells, neutrophils, macrophages, and dendritic cells. The close association between certain genes, especially FATP5, and the presence of immune subsets that infiltrate tumor may indicate their importance in immune dysregulation in HCC. The SCD gene encodes an enzyme involved in the biosynthesis of fatty acids, primarily oleic acid. Cancer cells are resistant to chemotherapy-induced apoptosis partly because of the expression of SCD, which is mediated by phosphatidylinositol three kinase/c-Jun N-terminal kinases activation (Bansal et al., 2014). NAFLD fibrosis is known to be associated with an increase in CCL20, an essential inflammatory mediator (Chu et al., 2018). A poor prognosis is related to CCL20 expression in hepatocellular carcinomas after curative resection of cancer (X. Ding et al., 2012).

The GPAT3 (AGPAT9) gene encodes a lysophosphatidic acid acyltransferase family member. The protein encoded by this gene catalyses the conversion of glycerol-3-phosphate to lysophosphatidic acid in triacylglycerol synthesis (J. Cao et al., 2006). Mice with severe congenital generalised lipodystrophies exhibit insulin resistance and hepatic steatosis when GPAT3 is deficient (Gao et al., 2020). It was found that knocking down GPAT3 effectively inhibited HCC cell growth, induced cell apoptosis, and blocked mTOR signalling in HCC cells.

IL1RN encodes an antagonist protein (IL1RA) that binds to IL-1 as a natural antagonist. IL1RN is involved in developing NAFLD features (M. G. Wolfs et al., 2015). A serum level of L-1RA is associated with inflammation of the liver and higher levels of ALT regardless of obesity, alcohol consumption, or insulin resistance. There is potential for IL-1RA to be used as a non-invasive indicator of NASH inflammatory responses (Pihlajamäki et al., 2012).

PLIN1, an adipocyte-specific protein encoded by this gene, coats lipid storage droplets to protect them until hormone-sensitive lipases can break them down. In adipocytes, PLIN1 is the major cAMP-dependent protein kinase substrate, and it may inhibit lipolysis when unphosphorylated (J. H. Sohn et al., 2018). NAFLD (non-alcoholic steatohepatitis, NASH) leads to an upregulation of PLIN1. However, it impairs glucose homeostasis and may be protective against lipotoxicity33 (Carr and Ahima, 2016). Our study indicated that PLIN1 mRNA expression is positively linked to overall survival.

## 5 Conclusion

Generally, by utilising biological information research methods, we have identified six key genes for diagnosing NAFLD and HCC with MRFs. Moreover, five key genes were identified for the prognosis of HCC changes and the created gene marker composed of these genes was FABP5, CCL20, and GPAT3 may be the critical dangerous prognostic genes of HCC. PLIN1 and IL1RN are protective prognostic genes of HCC. Nevertheless, since our research is based on data analysis, further experiments would be required to confirm our findings. Nevertheless, we hope that our research findings will contribute to improving the diagnosis and treatment of liver disease associated with NAFLD and HCC progression.

## Data Availability

The original contributions presented in the study are included in the article/supplementary material, further inquiries can be directed to the corresponding authors.
